# Exploring METRNL as a novel biomarker in sepsis: diagnostic potential and secretion mechanism

**DOI:** 10.1186/s40560-025-00780-4

**Published:** 2025-04-09

**Authors:** Tian-Ying Xu, Jing-Xin Zhao, Ming-Yao Chen, Zhu-Wei Miao, Zhi-Yong Li, Yong-Qing Chang, Yu-Sheng Wang, Chao-Yu Miao

**Affiliations:** 1 Department of Pharmacology, Second Military Medical University/Naval Medical University, Shanghai, China; 2 Department of Anesthetic Pharmacology, School of Anesthesiology, Second Military Medical University/Naval Medical University, Shanghai, China; 3 Faculty of Anesthesiology, Changhai Hospital, Second Military Medical University/Naval Medical University, Shanghai, China; 4 Department of Critical Care Medicine, Changhai Hospital, Second Military Medical University/Naval Medical University, Shanghai, China; 5 Department of Critical Care Medicine, Naval Medical Center of PLA, Shanghai, China

**Keywords:** METRNL, Sepsis, Biomarker, Endothelium, Toll-like receptor 4, Endoplasmic reticulum, Golgi

## Abstract

**Background:**

Sepsis is a life-threatening condition with a high mortality rate in intensive care unit (ICU). However, rapid and accurate diagnostic criteria are still lacking. This pilot study explored the role of METRNL as a novel biomarker for sepsis by focusing on its diagnostic potential and rapid secretion mechanism.

**Methods:**

METRNL levels were measured in cell and animal models of sepsis. Serum samples from 107 sepsis patients and 95 non-septic controls in ICU were collected. Diagnostic performance of METRNL, Procalcitonin (PCT) and C-reactive protein (CRP) were assessed using ROC analysis. Endothelial cell-specific Metrnl gene knockout mice (EC-*Metrnl*^*−/−*^ mice) were used to identify the source of METRNL secretion. Chemical inhibitors and RNA interference were used to explore the secretion pathways.

**Results:**

In lipopolysaccharide (LPS)-induced cell and mouse models of sepsis, METRNL levels significantly increased in a dose- and time-dependent manner. Similarly, in the cecal ligation and puncture mouse models, serum METRNL levels were elevated over time and correlated with sepsis severity. In animals, serum METRNL increased within 1 h post-modeling, preceding PCT and CRP. Clinically, sepsis patients had significantly higher serum METRNL levels. ROC analysis showed area under the curves [95% confidence intervals] of 0.943 [0.91–0.975] for METRNL, 0.955 [0.929–0.981] for PCT and 0.873 [0.825–0.921] for CRP. At the optimal cutoff value, METRNL (91.6%) exhibited relatively greater diagnostic specificity than PCT (88.4%) and CRP (69.5%). EC-*Metrnl*^*−/−*^ reduced majority of serum Metrnl levels in sepsis mouse models. Inhibition of the endoplasmic reticulum-Golgi (ER-Golgi) pathway through chemical inhibitors or RNA interference significantly reduced METRNL levels in the supernatant of sepsis cell models compared to control groups. Similar results were obtained with Toll-like receptor 4 (TLR4) and ERK inhibitors.

**Conclusions:**

This pilot study demonstrates that METRNL is a novel potential biomarker for sepsis with diagnostic capability comparable to that of PCT. Serum METRNL rapidly increased during the early phase of sepsis. Mechanistically, it mainly originates from the endothelium during sepsis, and TLR4-ERK signaling mediates the rapid secretion of METRNL via the classical ER-Golgi pathway in response to LPS stimulation.

**Supplementary Information:**

The online version contains supplementary material available at 10.1186/s40560-025-00780-4.

## Background

Sepsis is a life-threatening condition characterized by organ dysfunction caused by an uncontrolled response to infection, leading to high rates of morbidity and mortality [1, 2]. A recent report in The *Lancet* estimated approximately 48.9 million cases of sepsis worldwide in 2017, with 11 million related deaths, accounting for about 20% of all deaths globally [3]. In developed countries such as the United States, at least 1.7 million adults develop sepsis each year, with over 350,000 fatalities occurring during hospitalization [4]. In low- and middle-income countries, sepsis remains the leading cause of death among intensive care unit (ICU) patients, with mortality rates reaching up to 80% [5]. The World Health Organization has recognized sepsis as a major global health threat and has prioritized its prevention, diagnosis, and treatment, making it a significant concern for public health worldwide [6].

Despite increased attention given to sepsis, its mortality rate remains high, primarily due to the lack of rapid and accurate diagnostic criteria. Current clinical methods for early-stage diagnosis are limited. The diagnosis of sepsis is based on the Sepsis-3 diagnostic criteria issued jointly by the American Society of Critical Care Medicine and the European Society of Critical Care Medicine in 2016: infection combined with a Sequential Organ Failure Assessment (SOFA) score of ≥ 2 [1]. The SOFA score involves 12 items across six systems and requires laboratory test results, clinical medication doses, and neurological specialist scores. In summary, the infection confirmation and SOFA score needed for the diagnosis of sepsis make the process complex and time-consuming, which is not conducive to the early detection and treatment of sepsis [7]. Therefore, identifying biomarkers with independent diagnostic capabilities and early detection abilities is urgently needed.

Hundreds of sepsis biomarkers have been proposed and studied, among which procalcitonin (PCT) and C-reactive protein (CRP) are the most widely used and extensively researched to date [8]. PCT is the only biomarker included in the sepsis diagnosis and treatment guidelines and is used primarily to guide antibiotic therapy. Numerous studies suggest that it can also be utilized for the diagnosis of sepsis [1]. CRP is a traditional inflammatory screening marker, but its specificity for sepsis diagnosis is relatively low due to its elevation in various infections, stress conditions, and other diseases [9].

METRNL is a novel secreted protein homologous to the neurotrophin Meteorin, with approximately 40% amino acid identity. METRNL is also known as Meteorin-like, Meteorin-β [10], Cometin [11], Subfatin [12], or interleukin-41 [13]. METRNL plays essential roles in various processes, such as glucolipid metabolism [14, 15], cardiovascular diseases [16, 17], and tissue repair [18],etc. As early as 2016, using *Metrnl* knockout animals and recombinant METRNL protein, our laboratory discovered the protective role of METRNL in sepsis and applied for a Chinese patent [19]. Two additional studies also confirmed the important role of the METRNL in sepsis [10, 20]. Immune and inflammatory responses are closely related to the development of sepsis [21]. Several reports have shown that METRNL is related to early events of immune inflammation. For example, after stimulation with the type II cytokine IL-4, METRNL shows a significant increase in expression and secretion in the early stages [22]. Additionally, METRNL can act as an upstream cytokine to rapidly regulate the production of various cytokines and chemokines [10]. Thus, METRNL is probably involved in the early stages of sepsis. These findings prompted us to explore whether the METRNL can serve as a diagnostic biomarker for sepsis, and reveal its early response characteristics and secretion mechanism.

In the present study, we demonstrated serum METRNL elevation at the early phase in experimental sepsis models and validated METRNL as a potential biomarker for the diagnosis of sepsis in clinical patients. Additionally, we conducted a detailed comparison of METRNL with PCT and CRP in terms of their performance in diagnosing sepsis, as well as their response time in sepsis. Furthermore, the molecular mechanism of its secretion during sepsis, through which it can be used as a biomarker, was elucidated.

## Methods

### Animals

Male C57BL/6J mice were obtained from Shanghai Sippe-BK Lab Animal Co. Ltd (China), and endothelial cell-specific Metrnl gene knockout (EC-*Metrnl*^*−/−*^) mice were generated and identified as previously described [15, 23]. The primers used for identification are listed in Supplementary Table S1. The mice were housed under controlled conditions (temperature: 24 ± 2 °C; lighting: 8:00–20:00; relative humidity: 30–80%) with free access to standard animal chow and tap water. The animal experiments were conducted in accordance with the National Institute of Health Guide for the Care and Use of Laboratory Animals and were approved by the Institutional Animal Care and Use Committee of Naval Medical University (Shanghai, China).

### Cell culture and treatments

Primary human umbilical vein endothelial cells (HUVECs) were isolated and pooled from freshly delivered umbilical cords via established methods [24]. HUVECs were cultured in endothelial cell medium (ECM) (ScienCell, Carlsbad, CA, USA), and serum-free ECM was used when measuring METRNL levels in the cell supernatant. The 3rd to 5th passages of cells were used.

### Mouse model

Mice were intraperitoneally injected with different concentrations of lipopolysaccharides (LPS) (from *Escherichia coli* O111:B4, Sigma, St. Louis, MO, USA) to simulate the different severities of sepsis [25]. The cecal ligation and puncture (CLP) model was established according to the Nature Protocol [26]. In brief, mice were anesthetized, and the cecum was exteriorized through a midline laparotomy. The cecum was then ligated without obstructing the ileocecal valve and punctured with a single through-and-through puncture using a 21-gauge needle. A small droplet of fecal content was extruded to ensure patency. For most experiments, a moderate sepsis (mid-grade) model was used, in which the cecum was ligated at 50% of its length (measured from the distal pole to the base of the cecum). For the experiment comparing serum Metrnl levels under different sepsis severities, an additional severe sepsis (high-grade) model was employed, where the cecum was ligated at 75% of its length. Sham-operated mice underwent identical procedures, including anesthesia, laparotomy, and cecum manipulation, but without ligation or puncture. Postoperative care included subcutaneous administration of prewarmed saline (5 mL/100 g body weight) for resuscitation.

### Measurement of the METRNL, PCT, and CRP

METRNL levels in the supernatants of HUVECs, human serum, and mouse serum were detected via species-specific enzyme-linked immunosorbent assay (ELISA) kits (Human-DY7867/Mouse-DY6679, Bio-Techne, Minneapolis, MN, USA) according to the manufacturer's instructions. The PCT and CRP levels of patients were obtained directly from the hospital's medical records management system. The serum PCT and CRP levels in the mice were measured via ELISA according to the manufacturer's instructions (PCT: E-EL-M2419; CRP: E-EL-M0053, Elabscience, Wuhan, China).

### RNA Interference

RNA interference experiments targeting human SAR1A, SAR1B, and scrambled control siRNA were designed and synthesized by Hanbio (Shanghai, China). The sequences of the sense strands were as follows: *SAR1A* siRNA, forward: 5′-CCAAUGUGCCAAUCCUUAUTT-3′ and reverse: 5′-ACGUGACACGUUCGGAGAATT-3′; *SAR1B* siRNA, forward: 5′-CUACCUUCCUGCUAUCAAUTT-3′ and reverse: 5′-AUUGAUAGCAGGAAGGUAGTT-3′. siRNA transfections were performed via Lipofectamine RNAiMAX (Thermo Fisher Scientific, Waltham, MA, USA) according to the manufacturer’s instructions. The efficiency of siRNA knockdown was assessed via real-time reverse transcription polymerase chain reaction (RT-PCR).

### Western blotting

Western blotting was conducted as described in our previous study [27]. In brief, to assess p-ERK phosphorylation levels following stimulation with different concentrations of LPS, HUVECs were stimulated with different concentrations of LPS, and the cells were harvested 30 min later. For the U0126 inhibition experiment, HUVECs were pretreated with U0126 (10 μM) for 2 h, and then stimulated with LPS. The cells were harvested 2 h after the LPS stimulation. The harvested cells were then lysed with cell lysis buffer for Western and IP (Beyotime, Shanghai, China) containing protease and phosphatase inhibitor cocktail (Beyotime, Shanghai, China). Equal amounts of protein (15 μg) were separated via SDS-PAGE (Epizyme Biomedical Technology, Shanghai, China) and transferred onto PVDF membranes (Merck Millipore, Billerica, MA, USA). After being blocked for 3 h with 5% bovine serum albumin at room temperature, the membranes were incubated with the relevant primary antibodies. The primary antibodies used included the ERK1/2 antibody (Beyotime #AF1051), phospho-ERK1/2 antibody (Beyotime #AF1891), and tubulin antibody (Beyotime #AF2827). The membranes were subsequently incubated with an HRP-conjugated secondary antibody (Cell Signaling Technology, Danvers, MA, USA). Proteins were detected by enhanced chemiluminescence (ECL). ImageJ was used to quantify the intensity of the protein bands [28].

### Study subjects and sample collection

This study was conducted in the ICUs of Shanghai Changzheng Hospital (China) and Shanghai Changhai Hospital (China), enrolling adult patients (aged ≥ 18 years) who met the Sepsis-3 criteria in the first 24 h of ICU admission. Patient data, including the SOFA score, CRP content, PCT content, infection sites, microbial culture results, and death in 28 days were extracted. The exclusion criteria included patients with recent stroke and those with ulcerative colitis (as both conditions have been shown to significantly affect serum METRNL levels, based on prior research conducted in our laboratory and published studies). The control group consisted of adult ICU patients without sepsis, with the same exclusion criteria applied. This study enrolled patients in the sepsis patient group (n = 107), whereas those without sepsis were treated as controls (n = 95). The study was approved by the Committee on Ethics of Medical Research, Second Military Medical University/Naval Medical University, and conducted in accordance with the principles outlined in the Declaration of Helsinki. Written informed consent was obtained from patients or legal representatives.

For patients with sepsis, 2 mL of venous blood was collected within 24 h of sepsis diagnosis, either during routine blood collection or from an established deep venous catheter to avoid additional puncture. The SOFA score was determined on the day of blood collection, and 28 day survival was recorded. In the control group, 2 mL of venous blood was collected within the first 24 h of ICU admission, either at the same time as the routine blood collection or from an established deep venous catheter to avoid additional puncture. As one of the original control group patients later developed sepsis, we were able to collect blood samples from this patient both before and after the onset of sepsis. Within the limits of the ethical protocol, we closely monitored this patient and collected blood samples every other day until the patient’s death.

This study also included an additional analysis conducted in the ICU of Shanghai Changhai Hospital (China). Serum samples were collected from 24 patients undergoing major abdominal surgery, who were part of the previously mentioned control group. These patients had not developed sepsis at the time of preoperative and postoperative sample collection. Preoperative serum samples were collected within 24 h before surgery, and postoperative samples were obtained on the first postoperative day.

### Statistics

All the data are expressed as the means ± standard errors of the means (SEMs). Statistical analysis was performed with IBM SPSS Statistics for Windows v22.0 (IBM Corporation, Armonk, NY, USA) and GraphPad Prism v7.0 for Windows (GraphPad Software, Boston, MA, USA). Statistical differences between the two groups were assessed via Student’s t test, and differences across several groups were evaluated via the two-way analysis of variance (ANOVA). Receiver operating characteristic (ROC) analysis and logistic regression were applied to assess the diagnostic indicators' efficiency (sensitivity and specificity). *P* values less than 0.05 were considered statistically significant.

## Results

### Metrnl is elevated during the early stage of sepsis in experimental mouse models

**Fig. 1:**
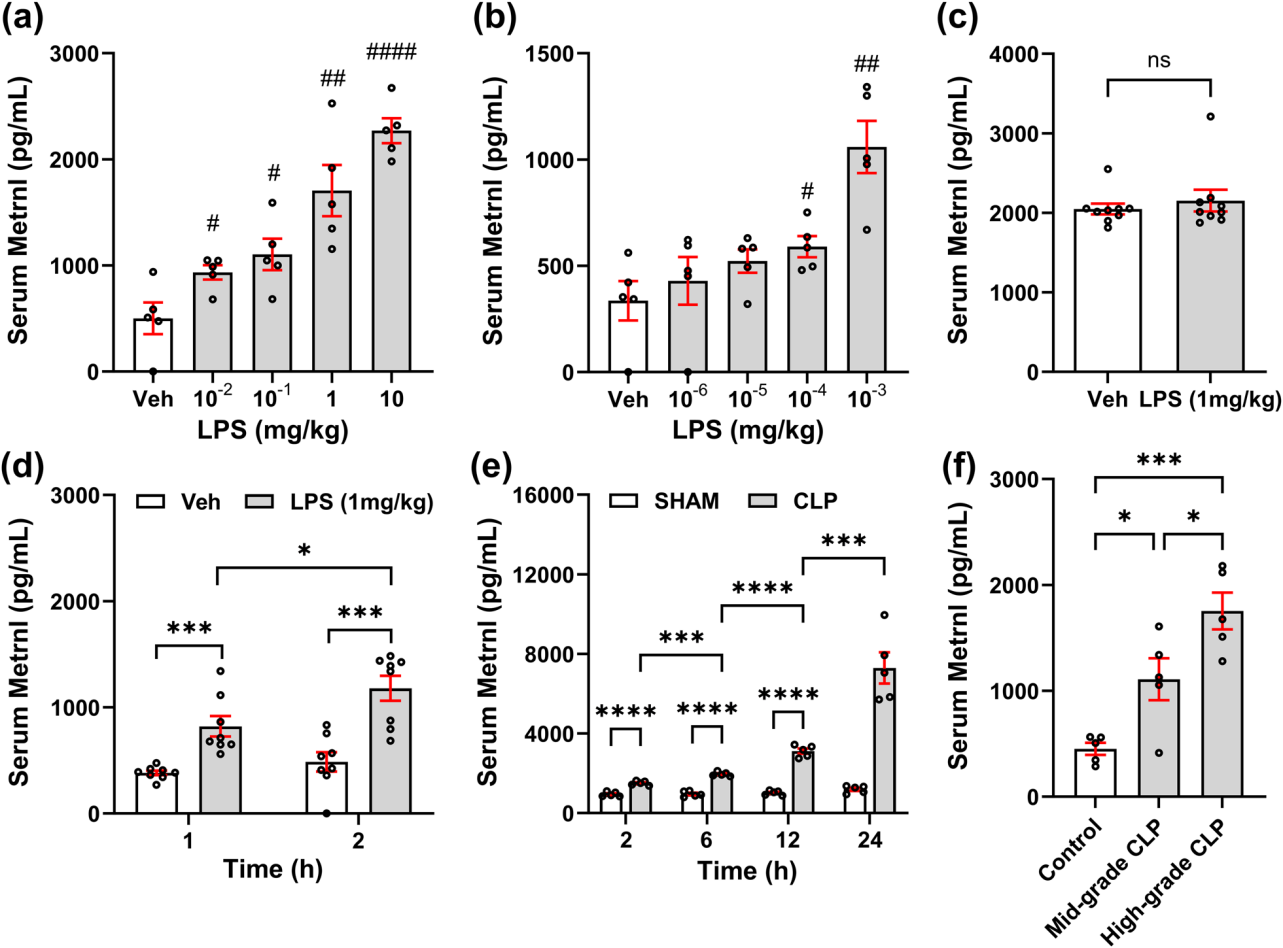
Metrnl is elevated during the early stage of sepsis in experimental mouse models. The mice were intraperitoneally injected with 10^–2^ to 10 mg/kg (**a**) or 10^–6^ to 10^–3^ mg/kg (**b**) of LPS or saline as a vehicle (Veh) control. Serum samples were collected 2 h later for Metrnl measurement. n = 5, ^#^*P* < 0.05, ^##^*P* < 0.01, ^####^*P* < 0.0001 vs. Veh; (**c**, **d**) Mice were intraperitoneally injected with 1 mg/kg of LPS or saline as a vehicle control. Serum samples were collected 0.5 (**c**), 1 or 2 h (**d**) later for Metrnl measurement. n = 8, ns: no significance, ^*^P < 0.05, ^***^P < 0.001; (**e**) Mice underwent CLP modeling, and serum samples were collected 2 to 24 h post-modeling for Metrnl measurement. n = 5, ^***^P < 0.001, ^****^P < 0.0001; (**f**) Mice underwent CLP modeling with different severity levels, and serum samples were collected 2 h post-modeling. The Control group was untreated. n = 5, ^*^P < 0.05, ^***^P < 0.001

To investigate the relationship between sepsis and Metrnl levels, we utilized an LPS-induced sepsis model. Initially, different doses of LPS were administered to simulate different severities of sepsis. Two hours postmodeling, the experimental groups treated with LPS doses ranging from 10^–2^ to 10 mg/kg presented behavioral manifestations, including hypovolemia, fatigue, slow movement, and observable hemoconcentration during blood collection. As depicted in Fig. 1a, the serum levels of Metrnl increased in a dose-dependent manner with increasing LPS dosage. Additionally, there was a significant difference in the serum Metrnl levels between all the LPS groups and the Veh group. Further exploration with lower doses of LPS gradients revealed that doses as low as 10^–4^ mg/kg could still increase serum Metrnl levels (Fig. 1b).

To investigate the diagnostic performance of Metrnl in sepsis, we examined the temporal relationship of serum Metrnl levels following the administration of a fixed dose of LPS (1 mg/kg). At 30 min postmodeling, there was no significant difference in the serum Metrnl levels between the LPS and Veh groups (Fig. 1c). However, as time progressed, a significant difference in the serum Metrnl levels between the LPS group and the Veh group emerged at 1 h postmodeling, and this difference further increased as time increased to 2 h (Fig. 1d).

We also employed a CLP-induced sepsis model, which better recapitulates the intricate progression of sepsis. Serum Metrnl levels were assessed at 2 to 24 h post-CLP modeling (Fig. 1e). At each time point, the serum Metrnl levels in the CLP group were consistently greater than those in the SHAM group. Moreover, with prolonged duration, the serum Metrnl levels in the CLP group tended to increase. Additionally, we compared serum Metrnl levels in mice subjected to different severity levels of CLP (Fig. 1f). Serum samples were collected 2 h post-CLP modeling. The serum Metrnl levels in the High-grade CLP group were significantly higher than those in the Mid-grade CLP group, suggesting a positive correlation between serum Metrnl levels and sepsis severity in the CLP model.

### Serum METRNL has high diagnostic efficiency for sepsis in the ICU

To evaluate the potential of the METRNL as a biomarker, we compared the serum levels of METRNL, PCT, and CRP between patients with sepsis and non-sepsis controls. A total of 202 patients were recruited, including 107 sepsis patients and 95 non-sepsis patients. The clinical information of the participants is presented in Table 1. There were no significant statistical differences in sex or age distribution between sepsis patients and non-sepsis controls.

**Table 1:** Characteristics of patients with sepsis and controls

Characteristics	Sepsis patients (n = 107)	Controls (n = 95)	*P* value
Male/female sex	66/41	57/38	0.808
Age, years	66(23–91)	62(24–90)	0.061
PCT ng/mL	3.53(0.111–100)	0.168(0.02–4.6)	< 0.001
CRP mg/L	115(10–452)	32.3(0.5–211.41)	< 0.001
METRNL pg/mL	934.16(321.60–1853.76)	371.62(171.56–1470.95)	< 0.001
Main diagnosis, no. of patients			
Sepsis	107	NA	/
Multiple trauma	NA	42	/
Postoperative status of major abdominal surgery	NA	24	/
Cardiovascular and cerebrovascular diseases	NA	12	/
Digestive system diseases	NA	8	/
Others	NA	9	/
Infection site, no. of patients			
Respiratory	43	NA	/
Abdominal	34	NA	/
Urinary	13	NA	/
Vascular	9	NA	/
Other	8	NA	/
Isolates, no. of patients			
Gram positive	22	NA	/
Gram negative	64	NA	/
Miscellaneous	21	NA	/
SOFA score	6(2–19)	2(1–3)	/
Died/survived	17/90	9/86	/

Data are expressed as median (interquartile range) unless otherwise indicated

**Fig. 2:**
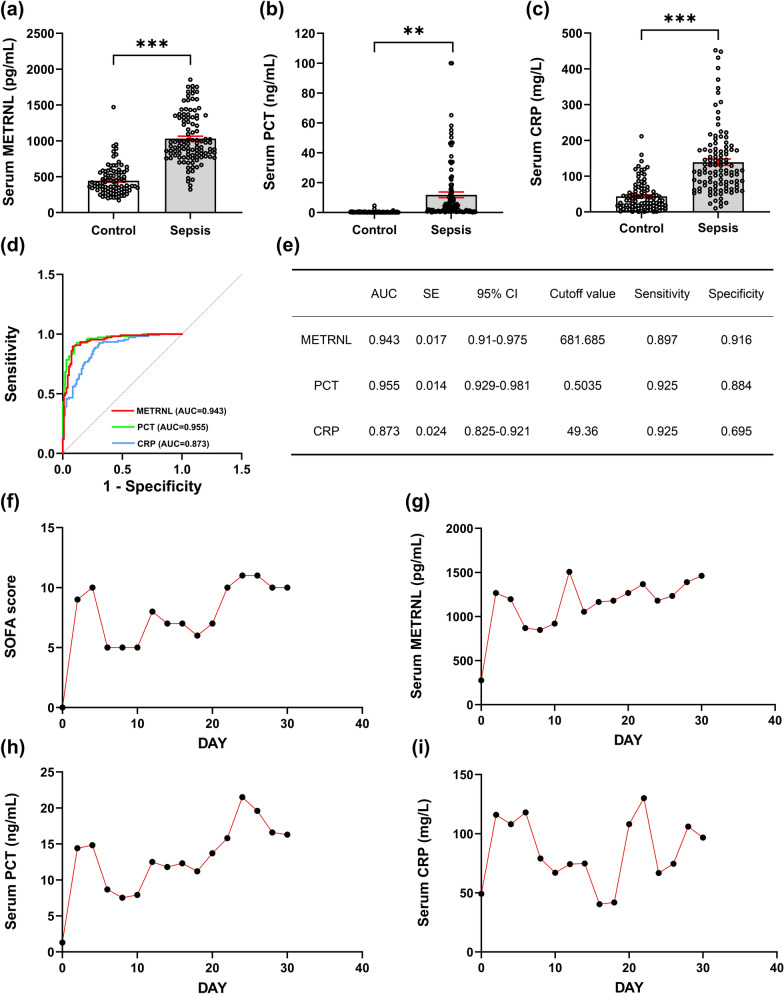
Serum METRNL has high diagnostic efficiency for sepsis in the ICU. Comparison of serum METRNL (**a**), PCT (**b**), and CRP (**c**) levels between sepsis patients (n = 107) and control patients (n = 95). ^**^P＜0.01, ^***^P＜0.001; (**d**) AUC of serum METRNL, PCT, and CRP for sepsis diagnosis; (**e**) Diagnostic performance of METRNL, PCT and CRP for sepsis diagnosis. The percentage of sepsis patients detected positive by each biomarker was calculated to show the sensitivity, which was 0.897 (96/107) for METRNL, 0.925 (99/107) for PCT and 0.925 for CRP (99/107). The percentage of non-sepsis patients detected negative by each biomarker was calculated to show the specificity, which was 0.916 (87/95) for METRNL, 0.884 (84/95) for PCT and 0.695 (66/95) for CRP; The continuous changes of SOFA score (**f**), serum METRNL (**g**), PCT (**h**), and CRP (**i**) before and after sepsis in a single patient. Day 0 represents the time before sepsis onset, followed by data collection every 2 days

As shown in Fig. 2a–c, the serum levels of METRNL, PCT, and CRP were increased in patients with sepsis in the ICU. Specifically, the mean value of METRNL increased from 443.18 ± 20.95 in control patients to 1031.16 ± 33.59 in sepsis patients, representing an increase of approximately 2.33 times. The diagnostic ability of biomarkers is typically evaluated via receiver operating characteristic (ROC) curves, where a larger area under the curve (AUC) indicates greater diagnostic accuracy. ROC curves for METRNL, PCT, and CRP analysis (Fig. 2d) revealed that METRNL and PCT had similarly high diagnostic accuracies for diagnosing sepsis, whereas CRP was less efficient (PCT: AUC 0.955, 95% CI 0.929–0.981; METRNL: AUC 0.943, 95% CI 0.91–0.975; CRP: AUC 0.873, 95% CI 0.825–0.921). Generally, the maximal Youden index determines the optimal diagnostic cutoff value [29]. In our study, the optimal cutoff values for METRNL, PCT, and CRP were determined to be 681.685 pg/mL, 0.5035 ng/mL, and 49.36 mg/L, respectively (Fig. 2e). At the optimal cutoff value, the sensitivities of METRNL, PCT, and CRP were all approximately 0.9. In terms of specificity, both the METRNL and PCT were also approximately 0.9, whereas the CRP was less than 0.7. Generally, METRNL and PCT demonstrated superior diagnostic performance, whereas CRP showed relatively poor diagnostic performance because of its lower AUC value and specificity.

In addition, we were fortunate to collect continuous data from a sepsis patient spanning 30 days (Fig. 2f–i), with samples collected every two days, covering the period from pre-sepsis to post-sepsis onset. These longitudinal data provide a better understanding of the changes in various biomarkers during the progression of sepsis. The patient did not have sepsis on day 0. Two days later, the SOFA score indicated that the patient had developed sepsis, and at that time, the patient's serum METRNL, PCT, and CRP levels were approximately 4.5, 11, and 2.3 times the pre-sepsis levels, respectively. Additionally, the METRNL and PCT levels remained consistently elevated throughout the progression of sepsis, whereas the CRP level temporarily decreased below the baseline.

### Serum Metrnl increases earlier than PCT and CRP in the LPS-induced sepsis model

**Fig. 3:**
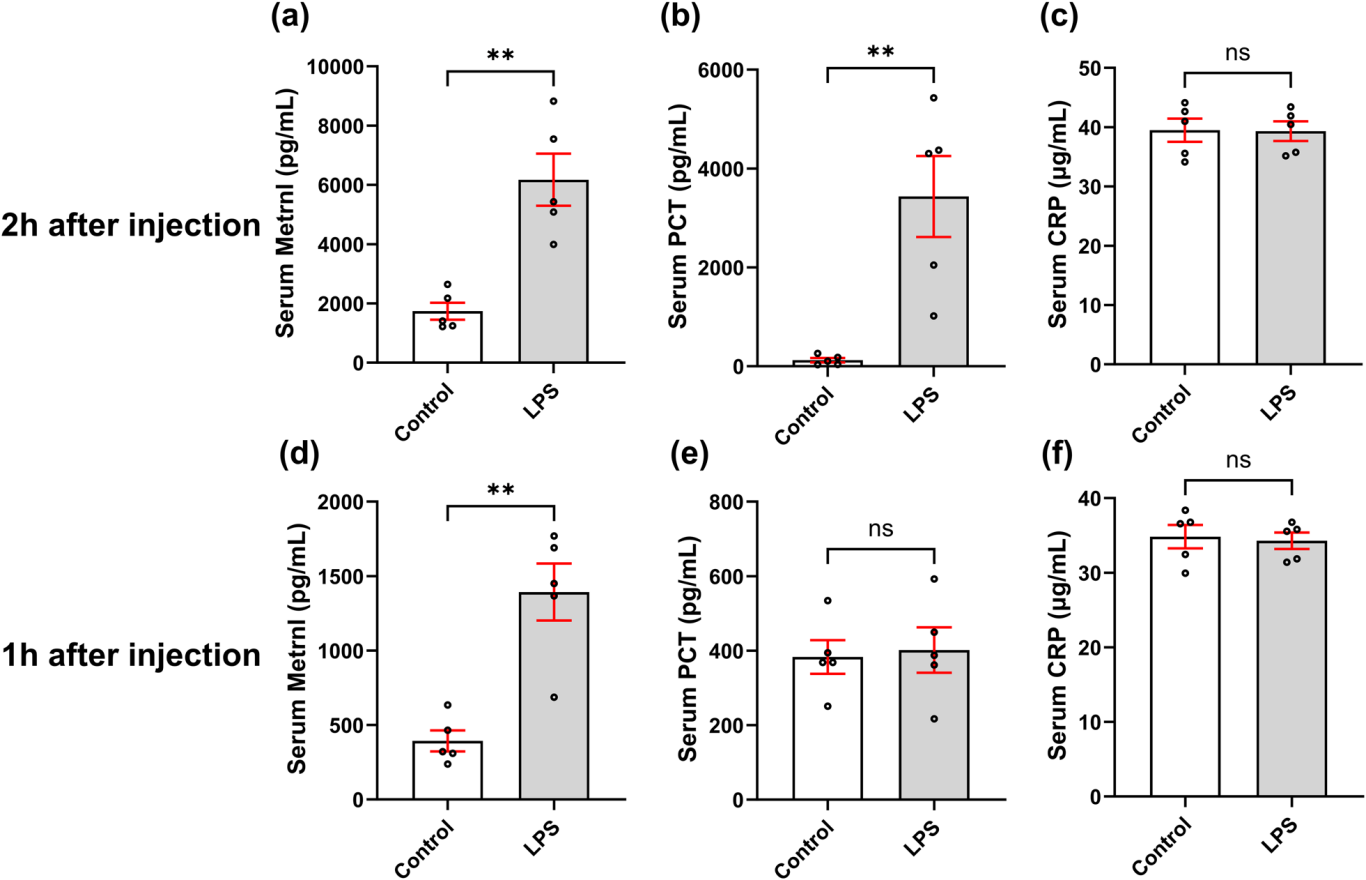
Serum Metrnl rises earlier than PCT and CRP in LPS-induced sepsis model. The mice were intraperitoneally injected with 1 mg/kg of LPS or saline as a control and serum samples were collected 2 h (**a**–**c**) or 1 h (**d**–**f**) after injection. n = 5, ns: no significance, ^**^P < 0.01

To further validate the potential of Metrnl as a biomarker for sepsis, we conducted a comparative analysis between Metrnl and established sepsis biomarkers in animal models. Two hours after LPS-induced sepsis modeling, the results revealed a significant increases in the serum Metrnl and PCT levels compared with those in the control group, whereas the serum CRP levels remained unchanged (Fig. 3a–c). Subsequently, investigations were also carried out at the 1 h post-modeling time point. Compared with those in the control group, the serum PCT and CRP levels were not significant different, whereas the Metrnl levels continued to exhibit a significantly increase (Fig. 3d–f). These findings indicate that, compared with classical markers such as PCT and CRP, Metrnl has greater sensitivity and responsiveness in the early stages of sepsis.

### Serum METRNL is derived mainly from endothelial cells under septic conditions

**Fig. 4:**
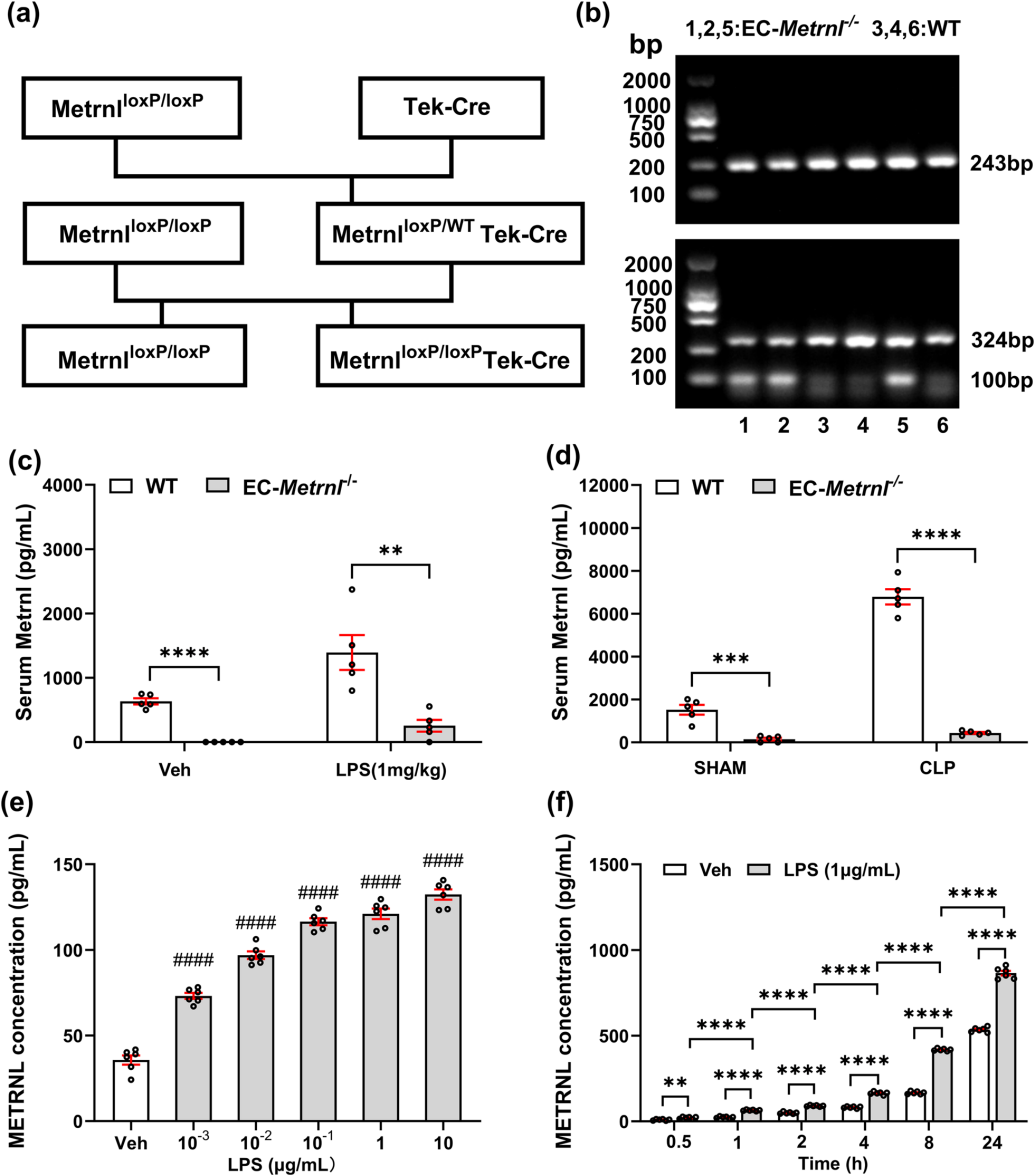
Serum METRNL is derived mainly from endothelial cells under septic conditions. (**a**) Endothelial cell (EC)-specific *Metrnl* knockout (EC-*Metrnl*^−/−^) mice were generated by mating *Metrnl*^*loxP/loxP*^ and *Tek-Cre* mice; (**b**) Identification of the EC-*Metrnl*^*−/−*^ mice. 1, 2, 5: *Metrnl*^*loxP/loxP*^* Tek-Cre* (EC-*Metrnl*^*−/−*^); 3, 4, 6: *Metrnl*^*loxP/loxP*^ (WT); The PCR products of *Metrnl*^*loxP*^, *Tek-Cre*, and the internal reference gene are 243 bp, 324 bp, and 100 bp, respectively; (**c**) EC-*Metrnl*^*−/−*^ mice and WT mice were intraperitoneally injected with 1 mg/kg LPS or saline as Veh control. Serum samples were collected 2 h after injection; (**d**) EC-*Metrnl*^*−/−*^ mice and WT mice underwent CLP modeling or sham surgery. Serum samples were collected 2 h after modeling. n = 5, ^**^P < 0.01, ^***^P < 0.001, ^****^P < 0.0001; (**e**) HUVECs were stimulated with 10^–3^ to 10 mg/mL LPS or Veh control for 2 h, and the cell supernatants were collected for METRNL measurement. n = 6, ^####^*P* < 0.0001 vs. Veh; (**f**) HUVECs were stimulated with 1 mg/mL LPS or vehicle control for 0.5 to 24 h, and cell supernatants were collected for METRNL measurement. n = 6, ^**^P < 0.01, ^****^P < 0.0001

To determine whether serum METRNL is secreted primarily by endothelial cells under septic conditions, mice with endothelial cell-specific knockout of *Metrnl* (EC-*Metrnl*^*−/−*^) were generated by mating *Metrnl*^*loxP/loxP*^ mice with Tek-Cre mice (Fig. 4a, b). As shown in Fig. 4c, the serum Metrnl levels in EC-*Metrnl*^*−/−*^ mice in the Veh group were significantly lower than those in WT mice, and the serum Metrnl level was below the detection limit, suggesting that the serum Metrnl secretion is derived mainly from the vascular endothelium under physiological conditions. Next, we investigated the primary source of serum Metrnl during sepsis in both the LPS-induced and CLP-induced sepsis mouse models. In the LPS-induced sepsis mouse model, the serum Metrnl level in the experimental group of EC-*Metrnl*^*−/−*^ mice was approximately 18% of that in WT mice (Fig. 4c). We subsequently established CLP sepsis models in EC-*Metrnl*^*−/−*^ and WT mice and blood samples were collected 24 h after modeling to detect serum Metrnl. All the mice exhibited hypovolemia and hemoconcentration. As shown in Fig. 4d, the serum Metrnl level in sham EC-*Metrnl*^*−/−*^ mice was approximately 10% of that in WT mice, and the serum Metrnl level in CLP-modeled EC-*Metrnl*^*−/−*^ mice was approximately 6% of that in CLP-modeled WT mice. Additionally, compared with those in the corresponding control groups, the mean serum Metrnl levels in WT mice increased by 757.94 pg/mL after LPS modeling and by 5273.27 pg/mL after CLP modeling. In contrast, the increases in those of EC-*Metrnl*^*−/−*^ mice were only 254.71 pg/mL and 288.26 pg/mL, respectively, indicating that the elevation of serum Metrnl levels in the sepsis model mice was significantly reduced after endothelial cell-specific knockout of *Metrnl*. These results indicate that the vascular endothelium is the primary cell source of serum Metrnl in the septic state.

Furthermore, we explored the dose–response and time-response relationships of METRNL levels in an LPS-induced sepsis cell model. Initially, HUVECs were treated with different concentrations of LPS, and METRNL levels in the cell supernatant were uniformly measured 2 h post-treatment. As depicted in Fig. 4e, compared with the Veh group, the group treated with LPS at concentration as low as 10^–3^ μg/mL presented in nearly doubled METRNL levels in the cell supernatant. Furthermore, with increasing LPS incubation concentrations (from 10^–3^ to 10 μg/mL), the METRNL level in the cell supernatant increased in a dose-dependent manner. The cells were subsequently incubated with an equal concentration of LPS (1 μg/mL), and the METRNL levels in the cell supernatant were measured at different time points ranging from 0.5 to 24 h post-treatment. Treatment with LPS for 30 min induced a significant increase in METRNL in the supernatant. The METRNL levels in the LPS groups were significantly different from those in the corresponding Veh groups. A time-response relationship between METRNL levels and the duration of LPS exposure was also observed (Fig. 4f).

### METRNL is secreted via the classical ER-Golgi pathway under LPS stimulation

**Fig. 5:**
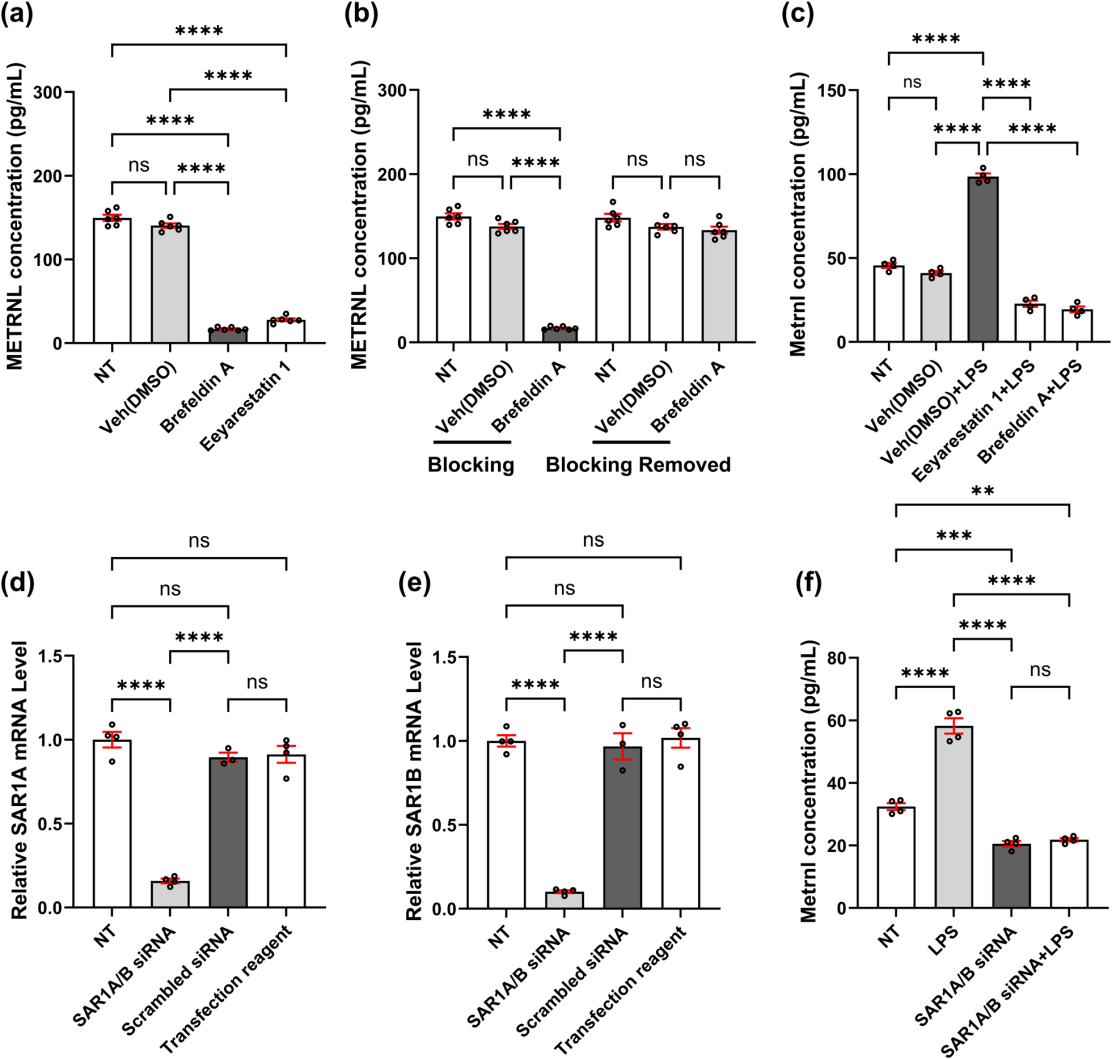
METRNL is secreted via the classical ER-Golgi pathway under LPS stimulation. (**a**) METRNL secretion from primary cultured HUVECs was reduced by Eeyarestatin 1 (5 mg/mL) or Brefeldin A (5 mg/mL). n = 6; (**b**) Recovery of METRNL release from HUVECs after Brefeldin A removal. Brefeldin A (5 mg/mL) or a vehicle control (DMSO) was added to the serum-free culture medium 2 h before cell supernatant collection for METRNL measurement. Then the cells were washed for three times and incubated in normal culture medium without drugs for 16 h followed by another 2 h of incubation in serum-free medium before METRNL detection. n = 6; (**c**) METRNL secretion from HUVECs induced by LPS (1 mg/mL) stimulation was inhibited by Eeyarestatin 1 (5 mg/mL) or Brefeldin A (5 mg/mL). n = 4; (**d**, **e**) After transfection with SAR1A/B siRNA, the mRNA expression of SAR1A (**d**) and SAR1B (**e**) was reduced in HUVECs; (**f**) METRNL secretion from HUVECs induced by LPS (1 mg/mL) stimulation was reduced by transfection with SAR1A/B siRNA. n = 4. ns: no significance, ^**^P < 0.01, ^***^P < 0.001, ^****^P < 0.0001

In the classical endoplasmic reticulum-Golgi (ER-Golgi) pathway, proteins are transported from the endoplasmic reticulum to the Golgi apparatus via COP II vesicles, and all three components are essential [30–32]. First, we used two inhibitors (Brefeldin A and Eeyarestatin 1) to validate the involvement of the endoplasmic reticulum and Golgi apparatus in the secretion of METRNL under LPS stimulation. Brefeldin A blocks the transfer of newly synthesized proteins from the endoplasmic reticulum to the Golgi apparatus [33]; Eeyarestatin 1 inhibits the construction of ER membrane transport channels [34]. In the Brefeldin A group, the METRNL levels in the HUVEC supernatant were approximately 10% of those in the solvent control group, whereas in the Eeyarestatin 1 group, the METRNL levels were approximately 20% of those in the solvent control group (Fig. 5a). Additionally, upon removal of Brefeldin A, the release of METRNL from endothelial cells was fully restored (Fig. 5b). We further applied these two inhibitors under septic conditions. The cells were treated with LPS and Eeyarestatin 1 or Brefeldin A for 2 h. As shown in Fig. 5c, the level of METRNL in the supernatant significantly increased in the Veh + LPS group. Moreover, treatment with Eeyarestatin 1 or Brefeldin A both blocked the LPS-induced increase in METRNL levels in the supernatant. These results indicated that the endoplasmic reticulum and Golgi apparatus were involved in the secretion of METRNL under LPS stimulation.

Second, we investigated the involvement of COP II vesicles. The small G protein SAR1, which contains two isoforms, SAR1A and SAR1B, is a critical component in the formation of COP II vesicles [35]. Therefore, we transfected HUVECs with *SAR1A/B* siRNA, which targets the two different isoforms of SAR1, to validate whether COP II vesicles are involved in METRNL secretion under septic conditions. RT-PCR revealed that the *SAR1A* knockdown efficiency was approximately 84.18% (Fig. 5d), and the *SAR1B* knockdown efficacy was approximately 89.88% (Fig. 5e). Transfection of *SAR1A/B* siRNA in HUVECs blocked the increase in METRNL levels in the supernatant induced by LPS and resulted in a decrease in the secretion levels of METRNL under normal conditions. (Fig. 5f). These results collectively demonstrated that METRNL was primarily secreted via the classical ER-Golgi pathway under LPS stimulation.

### LPS stimulation promotes the secretion of METRNL through TLR4 activation of downstream ERK kinase.

**Fig. 6:**
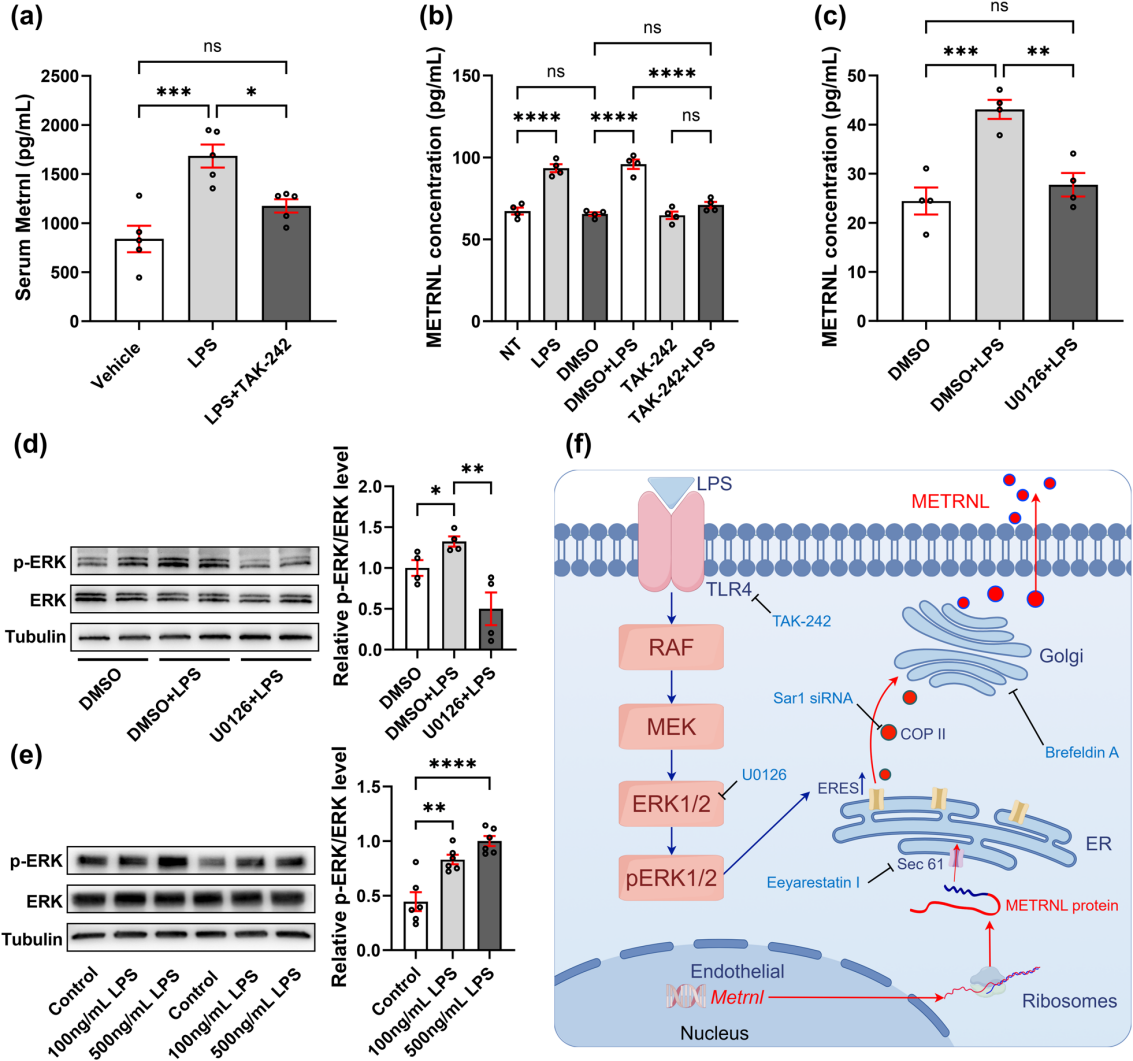
LPS stimulation promotes the secretion of METRNL through TLR4 activation of downstream ERK kinase. (**a**) TAK-242 reduced serum Metrnl increase induced by LPS modeling. Mice were pretreated with an intraperitoneal injection of TAK-242 (3 mg/kg) for 2 h, followed by an intraperitoneal injection of LPS (1 mg/kg). Serum were collected 2 h after injection. n = 5; (**b**) The increase in METRNL secretion from HUVECs induced by LPS (1 mg/mL) stimulation was inhibited by pretreatment with TAK-242 (1 μM) for 2 h. n = 4; The increase in METRNL secretion (**c**) and phosphorylation (**d**) from HUVECs induced by LPS stimulation (1 mg/mL) was inhibited by pretreatment with U0126 (10 μM) for 2 h. n = 4; (**e**) The levels of ERK phosphorylation in HUVECs increased with higher concentrations of LPS. ERK phosphorylation levels were measured 30 min after stimulation with LPS. n = 6; (**f**) Secretion and regulatory mechanism of METRNL in sepsis condition (By Figdraw). ns: no significance, ^*^P < 0.05, ^**^P < 0.01, ^***^P < 0.001, ^****^P < 0.0001

Toll-like receptor 4 is known for recognizing LPS and promoting inflammation by initiating the release of cytokines and chemokines [36–38]. To investigate the mechanism of LPS-induced METRNL release, we utilized TAK-242 [39, 40], a Toll-like receptor 4 (TLR4) inhibitor, to treat both animals and cells. In mice, TAK-242 (3 mg/kg) was intraperitoneally injected 2 h before LPS modeling, and blood samples were collected from the heart 2 h after modeling to measure serum Metrnl levels. As shown in Fig. 6a, the increase in mouse serum Metrnl induced by the intraperitoneal injection of LPS was reduced by TAK-242. Consistent with the results from the animal experiments, pretreatment of HUVECs with TAK-242 (1 μM) for 2 h followed by LPS administration and further incubation for 2 h resulted in significantly lower levels of METRNL in the cell supernatant in the TAK-242 + LPS group than in the DMSO + LPS group, indicating that TAK-242 blocked the LPS-induced increase in METRNL in the cell supernatant (Fig. 6b).

As an important group of signal transduction proteins, the ERK family regulates crucial processes such as cell growth, development, division, and death [41]. It has been reported that ERK is associated with protein secretion [42]. Therefore, we used the ERK phosphorylation inhibitor U0126 to investigate whether ERK acts as a key node protein connecting the TLR4 pathway and the ER-Golgi secretion pathway. The results revealed that using U0126 (10 μM) significantly reduced the increase in METRNL secretion induced by LPS stimulation (Fig. 6c), whereas a decrease in ERK phosphorylation was observed (Fig. 6d). Additionally, the phosphorylation of ERK increased with increasing doses of LPS (Fig. 6e). Overall, we concluded that the LPS-stimulated increase in METRNL secretion was mediated via the TLR4-ERK pathway.

## Discussion

In this study, we demonstrated an increase in the METRNL in both in vitro and in vivo sepsis models. In particular, the levels of METRNL were also elevated in the supernatants of cultured cells or the serum of mice in the early phase of sepsis modeling. Increases in the serum METRNL were further detected in sepsis patients. Compared with classical sepsis biomarkers such as PCT and CRP, the METRNL has earlier response kinetics and similar or greater diagnostic performance. We confirmed that the increased secretion of METRNL during sepsis primarily originates from endothelial cells. Mechanistically, we demonstrated that LPS stimulation activated downstream ERK kinases through TLR4, thereby promoting METRNL secretion through the classical ER-Golgi pathway (Fig. 6f).

Concurrently with our study, Chen et al. [20] investigated the role of METRNL in sepsis, and reported its potential as a diagnostic biomarker. However, there remains limited understanding of METRNL's performance and characteristics as a biomarker, and comparisons with existing markers are unclear. In our study, we compared the performance of METRNL with that of the most frequently used biomarkers, PCT and CRP, in ICU patients for the first time. Furthermore, we elucidated the rapid response characteristics of METRNL in detail and revealed its underlying secretion mechanisms in sepsis.

In terms of diagnostic performance, first, compared with PCT and CRP, METRNL can be detected at an earlier stage in sepsis models. At the cellular level, the level of METRNL in the supernatant of HUVECs significantly increased as early as 0.5 h after LPS incubation. This finding was further supported by the findings of animal experiments, where the earliest significant increase in serum Metrnl was observed at 1 h after LPS-induced sepsis in mice. However, the PCT and CRP levels remained unchanged at this time point. Second, we compared the diagnostic performance of the METRNL, PCT, and CRP in clinical trials for the first time. We found that under septic conditions, the serum METRNL level exhibited a relatively high fold change. For example, among 202 patients, the average value of the serum METRNL level in the sepsis group was 2.3 times greater than that in the non-sepsis group. Additionally, in continuous monitoring data of an individual patient, when first diagnosed with sepsis, the serum METRNL level was 4.5 times the pre-sepsis level. According to the ROC curve analysis, METRNL demonstrated a diagnostic performance similar to that of PCT and superior to that of CRP, with AUCs of 0.943 (0.91–0.975) for METRNL, 0.955 (0.929–0.981) for PCT and 0.873 (0.825–0.921) for CRP, respectively. At the optimal cutoff value, METRNL (0.916) exhibited relatively greater diagnostic specificity than PCT (0.884) and CRP (0.695). The diagnostic performance of PCT and CRP reported in our study is consistent with that reported in a meta-analysis of 28 clinical studies (PCT 0.91 [0.89–0.94]; CRP 0.85 [0.82–0.88]) [43]. The differences in the diagnostic performance of METRNL, PCT, and CRP may be related to variations in their secretion mechanisms and response speeds, which could influence their sensitivity and specificity. Serum METRNL is secreted primarily by vascular endothelial cells, rapidly responding to LPS stimulation via the TLR4-ERK signaling pathway and being released into the blood through the classical ER-Golgi secretion pathway. These mechanisms may partly explain METRNL’s significant elevation in serum levels during the early stages of sepsis (0.5–1 h) and its relatively high specificity observed in this study. In contrast, PCT secretion may be induced by pro-inflammatory cytokines, such as TNF-α and IL-6, involving multiple organs and tissues [44]. This systemic inflammatory response might enhance its sensitivity but could reduce its specificity (88.4%) compared to METRNL. CRP, on the other hand, is synthesized in the liver as part of the acute-phase response. Its secretion generally has a slower onset (6–12 h) and broader induction by various inflammatory stimuli [9], which might contribute to its lower specificity. These hypotheses provide a framework for future studies to further investigate the diagnostic potential of these biomarkers under different clinical conditions. On the basis of the above results, METRNL, with its good diagnostic performance, has the potential to be a candidate biomarker for sepsis diagnosis. The main conclusion on diagnostic performance drawn by Chen et al. [20] is similar to ours. The AUC level of METRNL in the ROC curve for diagnosing sepsis was lower than that in our study (0.80 vs 0.943). We speculate that this discrepancy is due to differences in clinical samples. For example, the median serum METRNL level in their clinical study of sepsis patients was less than 500 ng/mL, whereas in our study, it was 934.15 ng/mL. This suggests variations in disease progression among patients.

Inflammatory factors are significant contributors that may influence the specificity and diagnostic efficacy of biomarkers. According to existing studies and our laboratory’s previous research, serum METRNL levels tend to decrease in various chronic inflammation-related diseases, such as coronary artery disease [45], non-ST-segment elevation myocardial infarction [46] and ischemic stroke with chronic vascular disease [47]. In certain acute inflammatory conditions, such as major abdominal surgery, we conducted a preliminary study to evaluate the impact of these conditions on serum METRNL levels. Serum samples were collected preoperatively and on the first postoperative day. As shown in Supplementary Figure S1, serum METRNL levels increased slightly on the first postoperative day compared to preoperative levels, with a mean concentration 1.32 times higher postoperatively (446.83 pg/mL vs. 338.71 pg/mL). In contrast, the mean serum METRNL concentration in sepsis patients was 1031.16 pg/mL, 2.32 times higher than that of the control group (443.18 pg/mL). These findings indicate that while surgery has some impact on METRNL levels, the effect is modest compared to the substantial elevation observed in sepsis. While our study demonstrates the significant diagnostic potential of METRNL in sepsis, it is important to consider its broader role in inflammation. Existing literature presents conflicting findings regarding METRNL's relationship with cytokines. Chen et al. [20] suggest a negative correlation between METRNL and pro-inflammatory cytokines like TNF-α, IL-6, IL-8, and IL-1β, while other study indicate that METRNL secretion is upregulated in response to stimuli such as TNF-α, IL-4, IL-17, and IL-1β [22]. These discrepancies highlight the need for further studies to clarify whether METRNL is primarily indicative of sepsis or reflects a general inflammatory response. Other potential influencing factors like trauma, burns, and shock are needed to be evaluated with larger sample sizes. Such research will be crucial in determining METRNL’s diagnostic specificity and utility in clinical settings.

To better understand the rapid response process of METRNL during sepsis occurrence and provide more biological evidence for its potential as a diagnostic sepsis biomarker, we investigated the secretion mechanism of METRNL under septic conditions. METRNL secretion has been reported in HEK293 cells, COS-7 cells, HEK293F cells, 3T3-L1 adipocytes, primary adipocytes, HUVECs, adipose tissue, and gut tissue, etc. [11, 12, 23, 48, 49]. However, our previous research showed that the specific knockout of *Metrnl* in adipocytes, intestinal epithelial cells, or the liver did not significantly change the serum Metrnl levels, whereas endothelial cell-specific knockout significantly reduced serum Metrnl levels under physiological conditions [15, 23, 48, 49]. During sepsis, serum METRNL levels increase rapidly. Whether endothelial cells are also the major source of serum METRNL in sepsis remains to be confirmed. We utilized endothelial cell-specific *Metrnl* knockout mice to confirm for the first time that serum METRNL primarily originates from endothelial cells in sepsis. Moreover, even after endothelial-specific knockout of *Metrnl*, a small amount of METRNL secretion remained, suggesting the possibility of additional, though less significant, sources of METRNL secretion. It has been reported that METRNL secretion by bone marrow macrophages also increased under LPS stimulation [10], suggesting that the elevated serum METRNL levels during sepsis may be partially attributed to secretion from these cells.

Bioinformatic analysis of the METRNL protein sequence revealed that its N-terminus contains a signal peptide consisting of 45 amino acids [12, 48], which is required for secretion via the classical ER-Golgi pathway [50]. In the classical endoplasmic reticulum-Golgi (ER-Golgi) pathway, proteins are transported from the endoplasmic reticulum to the Golgi apparatus via COP II vesicles, and all three components are essential. Our previous research indicated that the endoplasmic reticulum and Golgi apparatus are involved in the secretion of METRNL under physiological conditions, but whether COP II is involved remains unclear. Therefore, whether METRNL is secreted via the classical ER-Golgi pathway under either septic or physiological conditions remains to be clarified. To investigate this, we stimulated HUVECs with LPS to simulate septic conditions and used Eeyarestatin 1 and Brefeldin A, both of which almost completely blocked the LPS-induced increase in METRNL, suggesting that the endoplasmic reticulum and Golgi apparatus are involved in METRNL secretion during sepsis.

In the classical ER-Golgi pathway, the COP II complex is responsible for forming vesicles at the ER exit sites (ERES), which mediate the cis-Golgi transport of proteins from the endoplasmic reticulum [51]. We further explored the role of COP II vesicles by using siRNA to inhibit their formation and found that LPS-induced METRNL secretion was completely inhibited, indicating that METRNL secretion during sepsis primarily relies on the classical ER-Golgi pathway. Additionally, we found that under physiological conditions, inhibiting COP II vesicles also led to a decrease in METRNL secretion levels. These findings, combined with our previous findings, demonstrate that under physiological conditions, METRNL secretion primarily occurs via the classical ER-Golgi pathway. However, we also noticed that under physiological conditions, using siRNA to knock down SAR1, thereby inhibiting the formation of COP II vesicles, resulted in approximately a 40% decrease in METRNL levels in the cell supernatant, without completely blocking its secretion. Therefore, it is possible that some METRNL may be secreted via an alternative non-classical pathway that bypasses COP II vesicles which needs further investigation.

We further explored the specific mechanism of the rapid LPS-induced secretion of METRNL. First, we propose for the first time that LPS can stimulate METRNL secretion via TLR4. In simple terms, when TLR4 on the cell surface is activated by LPS, it triggers MyD88- and TRIF-dependent pathways, leading to the production of pro-inflammatory cytokines to eradicate the infecting bacteria [52, 53]. Our research links METRNL to TLR4, identifying the downstream effectors of TLR4 and partly explaining the close relationship between METRNL and sepsis. Additionally, we identified ERK as a target protein downstream of TLR4 that promotes METRNL secretion. Studies have reported that 122 proteases or kinases, including ERK, can affect the transport of proteins from the endoplasmic reticulum to the Golgi apparatus, with ERK having the most significant impact [42]. Upon stimulation by upstream pathways, ERK is rapidly phosphorylated, increasing the number of ERES and thereby enhancing the transport of proteins from the ER to the Golgi. This finding likely explains the rapid secretion of METRNL in the early stages of sepsis. We also observed that the use of U0126 did not completely block METRNL secretion, suggesting the possible presence of additional targets influencing METRNL secretion beyond ERK. In addition, elucidation of the METRNL secretion mechanism and its important regulatory targets also provides potential therapeutic targets for sepsis and other diseases to achieve disease prevention and treatment through the regulation of endogenous METRNL secretion.

There are several caveats in this study. First, the clinical study within this research was designed as a pilot study with a relatively small sample size. Larger-scale and multicenter clinical studies are required to further evaluate the diagnostic role of METRNL in broader populations. Second, the clinical study did not include samples from the very early stages of sepsis (e.g., within the first 6–12 h post-onset). Future research should prioritize earlier sample collection to address this limitation. Third, even though we excluded patients with diseases such as stroke and ulcerative colitis, which are known to affect serum METRNL levels, it remains unclear whether serum METRNL levels change under other comorbid conditions given the diverse functions of METRNL, and further research is needed to clarify this.

## Conclusion

This study confirms the potential of METRNL as a novel biomarker for sepsis by demonstrating its ability to effectively diagnose sepsis and elucidate the rapid secretion mechanism of METRNL. The diagnostic performance of METRNL was comparable to that of PCT and superior to that of CRP. Compared with PCT and CRP, METRNL demonstrated better early responsiveness and greater diagnostic specificity. METRNL is secreted via the classical ER-Golgi pathway under both septic and physiological conditions, and the rapid secretion of METRNL under septic conditions is mediated by the TLR4-ERK pathway. These findings support further research to validate the clinical utility of METRNL and explore its biological role in sepsis, potentially leading to improved outcomes for sepsis patients through earlier intervention and targeted therapeutic strategies.

## Supplementary Information

Supplementary Material 1.

## Data Availability

The datasets during and/or analyzed during the current study available from the corresponding author on reasonable request.

## Abbreviations

PCT: Procalcitonin
CRP: C-reactive protein
ER-Golgi: Endoplasmic reticulum-Golgi
TLR4: Toll-like receptor 4
SOFA: Sequential organ failure assessment
HUVECs: Human umbilical vein endothelial cells
ECM: Endothelial cell medium
LPS: Lipopolysaccharides
CLP: Cecal Ligation and Puncture
ELISA: Enzyme-linked immunosorbent assay
RT-PCR: Real-time reverse transcription polymerase chain reaction
SEM: Standard error of mean
ANOVA: Analysis of variance
ROC: Receiver operating characteristic
AUC: Area under the curve
ERES: ER exit sites
