# Supplementary material for: Exploring METRNL as a novel biomarker in sepsis: diagnostic potential and secretion mechanism
Source: J Intensive Care. 2025 Apr 9;13:19. doi: 10.1186/s40560-025-00780-4 (PMC11983927; doi:10.1186/s40560-025-00780-4)
Supplement: Supplementary file 1 — Supplementary Material 1. [file 40560_2025_780_MOESM1_ESM.docx]

# Supplementary materials

**Table.S1 Primer sequences for identifying EC-*Metrnl^-/-^* mice.**

| Primer name | Forward primer (5′–3′) | Reversed primer (5′–3′) |
| --- | --- | --- |
| Metrnl-floxed | TGAGGGTTGGAGGCTCCTAGC | GGATGAGCGTTTGAGCACAGC |
| Tek-Cre | GCGGTCTGGCAGTAAAAACTATC | GTGAAACAGCATTGCTGTCACTT |
| Internal control | CTAGGCCACAGAATTGAAAGATCT | GTAGGTGGAAATTCTAGCATCATCC |


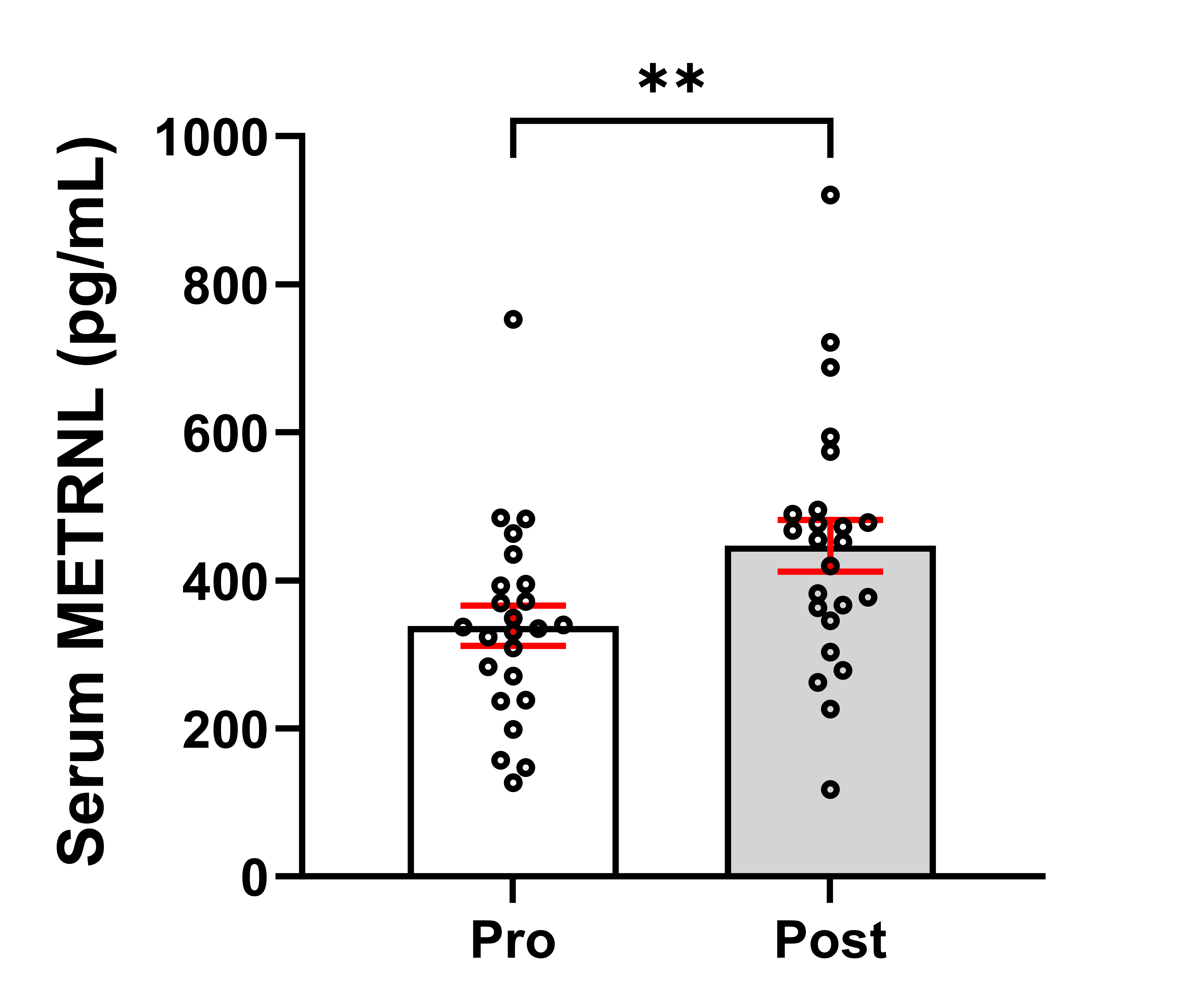


**Fig.S1 Changes in serum METRNL levels before and after major abdominal surgery.** Serum samples were collected preoperatively (within 24 hours before surgery) and on the first postoperative day in patients undergoing major abdominal surgery. n=24, ^**^*P*＜0.01.
